# Residual Creep Life Assessment of High-Temperature Components in Power Industry

**DOI:** 10.3390/s23042163

**Published:** 2023-02-14

**Authors:** Ivanna Pivdiablyk, Zhu Di Goh, Liam Kok Chye, Robert Shandro, Fabien Lefebvre

**Affiliations:** 1 Cetim-Matcor Technology and Services, 3 Seletar Aerospace Link, Singapore 797550, Singapore; 2 Cetim, 52 Avenue Félix Louat, 60300 Senlis, France

**Keywords:** high-temperature components, residual life assessment, creep, steel, power plants

## Abstract

A large percentage of power, petroleum, and chemical plants over the world were in operation for a long duration with the corresponding critical components being used beyond the design life of 30 to 40 years. It is generally more cost-effective to refurbish or modernize the degraded equipment or components, rather than to construct a new plant. Therefore, a reliable plant life extension assessment that can evaluate the critical components is needed. The key element in plant life extension is the residual life assessment technology. However, at present, there is still no general consensus among the industry players on the approach to adopt when performing residual life assessment for such a critical damage mechanism as creep. In this article, a three-level residual life assessment methodology is proposed as a general approach for high-temperature components prone to creep. A detailed validation of the selected guidelines and calculation models is also described. Eventually, an application of the three-level methodology to a real industrial case study is presented.

## 1. Introduction

Optimized overall life-cycle costs, availability, energy efficiency, and environmental performance are major objectives in a competitive power generation market [1]. There were many breakthroughs over the years in improving the above factors in the power generation industry through process and material innovations as well as from intelligent and cost-effective monitoring and assessment of the integrity and performance of the key components for power plants.

Residual life assessment (RLA) plays a major role in monitoring and life extension of components. The purpose of life extension activities is not to continue the operation of a plant beyond its useful life, but to ensure full utilization up to its useful life [2]. If RLA can be properly carried out to provide an accurate prediction of the service life, it will help to maximize a plant’s utilization by establishing a sound, defensible basis for extending operating life, reducing costly unscheduled outages caused by in-service failures, and eliminating unnecessary replacements [3].

Considerable research was carried out by various international bodies in developing new and refining existing RLA methodologies over the years [1,2,4,5,6,7,8]. Part of the reason is that there is still no consensus among the industry players on the approach to adopt when performing RLA for certain damage mechanisms. This is especially true for the power industry where creep damage is the critical and most systematic damage mechanism for components operating under elevated temperatures, such as boilers and heat recovery steam generators (HRSGs). The current state-of-the-art ultra-supercritical (USC) plants currently use steam at 600 °C to 620 °C, yet an advanced supercritical boiler (A-USC) was developed to operate at temperatures above 700 °C for better efficiency [9,10,11].

The boiler and HRSG sections that are susceptible to creep damage are the steam superheater (especially the high-pressure section), steam reheater, as well as the main steam piping [2]. Creep damage in headers tends to occur at the weldments, such as stub tube weld joints, longitudinal weld seams, header branch connections, or girth butt joints [2]. Such damage in weldments can generally be observed from the outer surface of the components in the form of cavities, cracks, or steam leak in extreme cases. In contrast, creep damage in the header base metal generally occurs by thermal softening with some extent of bulging damage prior to visible creep cavitation. Catastrophic rupture due to creep damage, apart from damage along the longitudinal weld seams, for header component is minimal.

Creep cracks in weldment can occur in the weld metal as well as the heat affected zone (HAZ) for different reasons. Creep cracks in weld metal are generally due to lower strength or lower ductility of the weld material compared to the rest of the components [2,12,13]. Creep cracks in HAZ region are essentially influenced by its microstructure and low strength, in which the creep resistance is generally the lowest as compared to the base metal and weld [14,15]. For steam piping containing longitudinal seam welds, such welds are generally the first location to have a steam leak due to creep damage. This is consistent with Cetim-Matcor’s experience, in which the discovery of creep damage was more commonly found in weld joints during numerous site inspections.

Although the above-mentioned components are designed to withstand a particular range of elevated temperatures and loads, any deviation of working environment from the design parameters may result in a shortened lifetime or, in a worst-case scenario, in unrepairable equipment with no possible maintenance [16]. As such, their residual creep life should be systematically monitored and calculated with different means, depending on the RLA approach adopted. Although creep resistance varies from one metallic alloy to another, it is considerably dependent on in-service temperature. The common materials that are used for superheater and reheater sections of HRSG and boiler, as well as main steam piping are Grade 22/22CrMo4-4/2.25Cr-1Mo, Grade 23/23CrMo 5/2.25CrMoVWNb, Grade 24/2.25Cr1MoVTiB, Grade 91/X10CrMoVNb9-1/9Cr-1Mo-V, and Grade 92/X10CrWMoVNb9-2/9Cr-2W.

After having reviewed and compared the assessment guidelines and calculation models used in macro-approaches for RLA in the market, including the current methodologies adopted by Cetim-Matcor, we propose an improvised methodology for RLA of static components prone to creep. It is based on the various approaches adopted by international bodies, such as the Energy Power Research Institute (EPRI), European Creep Collaborative Committee (ECCC), as well as established literature, journals, and books. The principal objectives are to determine the likelihood of creep damage based on the existing operating condition, the extent of damage through subsequent examinations, and to give recommendations on monitoring, detailed analysis, or replacement of studied components. The methodology is validated through the application to one of the Cetim-Matcor past case studies.

## 2. Review of Methodologies

Assessment of the residual life behavior of high-temperature components was always an area of interest for the industry. Creep damage is one of the primary failure mechanisms, and its complex nature demands particular attention for RLA. Therefore, several RLA methodologies were proposed by various corporations and laboratories.

### 2.1. ECCC Recommendations

Founded in 1991, ECCC developed and carried out several programs over the years, namely, development of recommendations for the generation, collation/exchange and assessment of creep data for virgin parent materials, assessment of creep data for weldments, expansion of the scope of data sheets to include the consideration of creep strain and ductility assessment, post exposed data, creep crack initiation data, and the analysis of high-temperature multi-axial features and components [17]. Nine recommendation volumes were published, among which, Volume 5: “Guidance for the assessment of uniaxial creep data” and Volume 9: “High temperature component analysis” specify RLA methodology or approaches [6,7].

The recommended RLA methodology in Volume 5 is not component specific and applies to all materials subjected to operating condition that are susceptible to creep damage [6]. It mainly focuses on the guidance for computation of the remnant life of serviced components based on both post exposure and virgin material creep data. It is the principal aim to ensure a credible extrapolation of the generally small and short time post-exposure data sets by applying rigorous pre-assessment, main assessment, and post-assessment recommendations. The following computational methods are selected for the assessment: parametric assessment—Larson Miller Parameter (LMP) and Manson Haferd Parameter (MHP), parametric assessment with linear damage accumulation rules, PD 6605, ISPESL procedure, original ENEL CRL procedure, and MPC Omega method.

Volume 9 deals with the review of current assessment procedures for components in terms of rupture and crack growth. It does not specify or recommend RLA methodology for creep damage [7]. The following assessment procedures are reviewed in this volume concerning creep damage: R5 (rupture), EN 12952-4, ISPESL, and TRD 508, and VGB-R509L.

### 2.2. EPRI Research in Power Generation Industry

Founded in 1972, EPRI published numerous papers on life extension of various systems in power generation industry, including boiler/HRSG, steam turbine and gas turbine systems. The available papers selected for review on the life extension assessment are: (1) “EPRI CS-4778 Generic guidelines for the life extension of fossil fuel power plants”, and (2) “EPRI CS-5588-V1 Remaining Life Estimation of Boiler Pressure Parts” [4,8].

The purpose of the first document is to present a procedural plan, which in conjunction with existing utility planning and technical procedures should ease the development of a life extension program [8]. These guidelines provide a typical road map for life extension activities and are supposed to apply to all systems related to the generation industry. A three-level approach is recommended in this paper. The key to this approach is to evaluate the component condition by levels, in which progressively more rigorous assessments are undertaken only if the component in question fails to demonstrate the desired residual life that is specified by the minimum criteria of the preceding step. For Level I assessment, two activities are required to be carried out, namely, to assemble elementary service factors and to answer critical screening questions for the component to be analyzed. If the information is sufficient to make a Level I assessment and if that level of assessment indicates that the calculated residual life is greater than or equal to the desired residual life, then the plant owner can move immediately to a regular inspection and periodic review program without additional work on that component. Level II assessment typically requires new information to be generated via an initial inspection, simplified stress analysis, measured dimensions or operating parameters, etc. If the residual life determined in Level II assessment is still less than the projected unit life, the more precise evaluation of Level III assessment is implemented, provided that the value of the component exceeds the cost of detailed evaluation.

According to the second document, the following information is necessary for life assessment: current dimensions, damage accumulations and strength of the component material, operating pressure, temperature, temperature gradient cycling conditions for future operation, and stress analysis incorporating damage accumulation models [4]. The RLA falls into two broad categories: category A involves the acquisition and monitoring of operational parameters and the use of standard material data, whereas category B is based on post-service examination and/or testing, which require direct access to the component for sampling and measurement. The RLA methodology in this paper is generally similar to the general guidelines in the document [8].

### 2.3. Damage Mechanisms and Life Assessment of High Temperature Components

The book by Viswanathan suggests a three-level approach to RLA for all components [2]. The described life assessment procedures are used only to ascertain that failure would not occur within the life extension period. The approach consists of the following:

1. Level 1: The assessment is performed using plant records, design stresses and temperatures, and minimum values of material properties from the literature.
2. Level 2: Actual measurements of dimensions and temperature, simplified stress calculations, and inspections coupled with the use of minimum material properties from the literature are required in this assessment.
3. Level 3: This assessment involves in-depth inspection, refined stress analysis, detailed plant monitoring records and the generation of actual material data from samples removed from the component.

The RLA approaches are further split into damage mechanism-specific, i.e., for bulk creep damage and for localised damage by crack growth, and component-specific, i.e., for outlet headers, steam pipes, and tubes in boilers and HRSGs, as well as rotors, casing, valves, and steam chests in steam turbines. The RLA approach for bulk creep damage can be classified into two broad categories: history-based and based on post-service evaluation of the actual components.

### 2.4. Power Plant Life Management and Performance Improvement

The life extension approaches that are proposed for the boiler and gas turbine systems by Oakey are generally similar, although no specific methodology is mentioned [1]. In the case of boilers/HRSGs, the RLA methods are generally split into two groups: the computational methods that collect the controlled operational parameters, standard material data, durability measurement and numerical assistance, and the methods that rely on non-destructive testing (NDT) or destructive (after-operation) testing. To increase the objectivity and reliability of RLA, both types of methods are generally adopted to evaluate the state of a material or component. Computational models can be used to assess the chambers, pipes, turbine rotors, and bodies following the requirements, e.g., EN-12952-4/2000, TRD-508, or ASME Code Case N-47. The Miner–Palmgren linear summation damage rule is used to calculate the residual life. It provides a rough estimate of accumulated damage or determination of the boiler components that should be subjected to more in-depth control.

### 2.5. Coal Power Plant Materials and Life Assessment

The three-level approach to RLA is also recognized and proposed as the best method for residual life estimation by Paddea et al. [5]. It comprises the use of three stages to assess the actual state of pressure equipment components:

1. Stage 1 is the calculation stage, based on the equipment history, taking into account operational pressure, temperature, incidental events, and the number of startups and shutdowns. It requires the compilation of relevant information, which can be obtained from technical drawings, original material list, material properties, database of operation conditions, and a detailed database of failures that occurred during the life of the equipment.
2. Stage 2 assessment involves NDT of the pressure components. This assessment can be improved from stage 1, because stage 1 can provide relevant information to select the best zone in which to perform NDT.
3. Stage 3 assessment relates to destructive testing. This assessment requires the removal of a small amount of material from the equipment. A comparison of the results of all tests performed is then required.

Such assessment procedures for the RLA calculation as R5 (rupture) approach, European norm EN 13445, EN 12952-4, and ISPESL 48/2003, TRD 508, and VGB R-509L are also introduced:

Based on the review of RLA approaches and assessment procedures provided by several papers, journals, and books from different organizations, all of them have the following similarities:

- The RLA assessment can be performed in either two-level or three-level approaches. Level I assessment is usually a preliminary residual life calculation using historical data. The results from this assessment can be used to plan for the inspection to be carried out on the critical components. Level II and/or III assessment usually involves NDT or destructive tests to improve the accuracy of the residual life calculation.
- A review of the history records of the components is required. If there is operating data available, the review can be performed using the operating records. If not, a preliminary review can be performed using the design data.
- RLA calculation/assessment procedures are carried out in both Level I and II assessments to obtain the residual life value of the components. There are various standards and guidelines available in the industry for the evaluation of the most appropriate models to be used for the components.
- The two-level or three-level approach applies to all types of systems in the power industry. However, the details of the assessment procedures will vary depending on the components and dominant damage mechanisms.

## 3. Proposed Methodology

**Figure 1:**
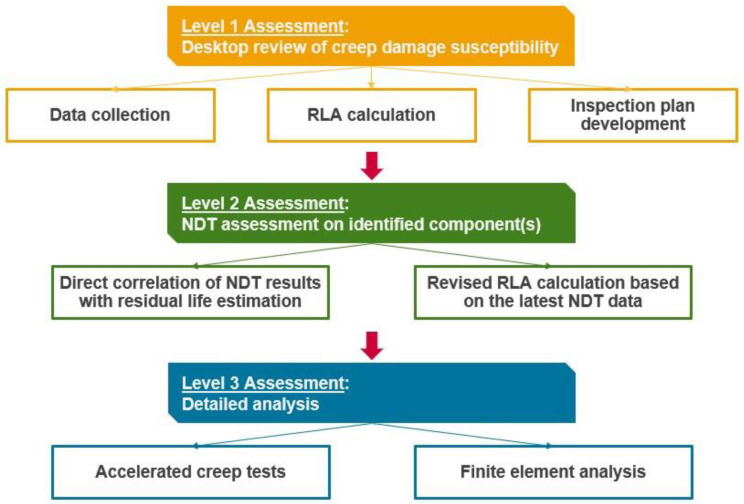
Proposed 3-level approach for creep RLA.

From the review, presented in the previous section, a three-level RLA approach (Figure 1) is proposed for the assessment of creep damage in pressurized equipment and components in boilers and HRSG, which are operating at a high-temperature environment. It is based on the integration of the relevant guidelines and good practices from the various procedures and literature reviewed.

The need for assessment solely at Level 1 (L1), or at L1 and Level 2 (L2), or at all three levels depends on whether a component is deemed to be susceptible to creep damage during L1 analysis. If the calculated creep life fraction consumed is significant in L1 assessment, then L2 assessment is recommended to ascertain the extent of creep damage. When the component is evaluated and it is found that it received some extent of creep damage, it is advised to proceed with Level 3 (L3) analysis. The assessment procedures are discussed in more detail in the following subsections. The application and validation of the proposed 3-level approach are performed on an industrial case study in Section 4.

### 3.1. Level 1

**Figure 2:**
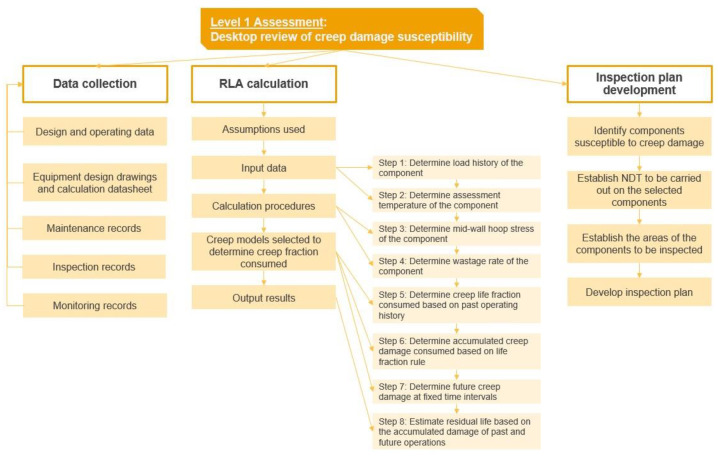
Flow chart on the steps for L1 approach in creep RLA.

L1 is a preliminary assessment based on the information available for review. The purpose is to estimate the creep life fraction consumed for the selected components based on the information available for review. The overall process for L1 is illustrated in the flow chart in Figure 2.

L1 approach comprises mainly three steps:

1. Data collection;
2. RLA calculations;
3. Inspection plan development.

#### 3.1.1. Data Collection

The data to be collected at the start of the assessment are split into five parts (Table 1). Duration of service, number of start-ups and shut-downs, material specifications, repair records, information from temperature sensors, strain gauges, etc., are some examples of the information required for L1 assessment.

Some of the information may not be available to perform the creep RLA calculation. In this case, assumptions are made to assist in the L1 assessment. The mandatory information required for L1 assessment, as well as the possible assumptions used for the calculation, are summarized in Table 1.

#### 3.1.2. RLA Calculation

Creep RLA calculation can be performed once the input data are available. It comprises 8 steps, detailed below. The assumptions made for creep life calculation procedures, especially for boiler tubes, are as follows [18]:

- The effect of the tube fins on the stress is ignored. Internal burst pressure tests on integral low-finned tubes show that the plain ends of a finned tube are its weakest points because the helical fins act as reinforcement rings.
- If the tube metal temperature is taken from the operation records (i.e., no internal oxide thickness measurement is available), the increase in the tube metal temperature resulting from the build-up of steam-side deposits or oxide scale is ignored.
- The creep properties of the component assessed are assumed to be similar to the data used for a different standard when calculating the creep life fraction consumed.

1. Step 1—determine the load history of the component

The load history refers to all significant loads and events applied to the components [19]. If the component is under cyclic operation, the load history should be divided into operating cycles. If the component is operating under base load conditions, the operating cycles can be identified based on the shutdown events.

Any significant variation in the operating cycle must be captured by several time increments. Provided that the component is subjected to erosion or corrosion, the time increment should be small enough to capture changes in the wall thickness. The thermocouple readings are required for establishing the temperature history of the superheater/reheater section if the temperature fluctuates throughout the service [20]. They also help to establish whether long-term overheating took place [21].

2. Step 2—determine the assessment temperature of the component
  The temperature data can be obtained from the following sources:
  ○ Thermocouples;
  ○ Maximum operating temperature data;
  ○ Metal temperature estimation based on internal oxide thickness measurement.
3. Step 3—determine the mid-wall hoop stress

The mid-wall hoop stress is computed according to Equation (1) for thin-wall and according to Equation (2) for the thick-wall components using elastic steady-state stress and steady-state creep solutions, respectively [18,19]. A safety factor of 1.3 is included in the hoop stress values to reflect the scatter and accuracy of the available material data [22].

σ_hoop_ = (P/2)(OD/t − 1), (1)

σ_1_ = C(1 + ((2 − n_BN_)/n_BN_)(R_0_/r)^2/n^_BN_)L_f_, (2)

P: operating pressure (MPa);
OD: outer diameter (mm);
t: measured thickness (mm);
n_BN_: Bailey–Norton coefficient;
R_0_: outer radius (mm);
L_f_: Lorentz factor.

4. Step 4—determine the wastage rate of the component

The component is subjected not only to creep damage but also to wall thinning due to oxidation and external corrosion. The wastage rate data are important, as continual metal loss results in an ongoing stress increase due to reduction in wall thickness. This will have an impact on the overall residual creep life calculation. The internal metal loss rate can be determined using measurements of oxide thickness. The latter is related to the metal wastage via the Pilling–Bedworth Ratio (PBR). When an oxide is formed at the metal/oxide interface, the volume change due to the formation of the oxide can be expressed as PBR by Equation (3) [23]. For the case of boiler tube, pipe and header, the main oxide formed is magnetite. The PBR for magnetite scale is 2.1 [24]. The wastage rate due to the formation of internal oxide is determined with Equation (4).

PBR = Volume of oxide/volume of metal,(3)

wastage rate = (1/PBR)t_oxide_/running hours, (4)

wastage rate: (mm/h);
t_oxide_: thickness of internal oxide (mm).

5. Step 5—determine the creep life fraction consumed by the component

Consumed creep life fraction (Equation (5)) is based on the past operation history represented by time increments.

Creep life fraction consumed = T_op_/T_rupture_, (5)

T_op_: running hours (h);
T_rupture_: time to rupture (h).

The time to rupture can be calculated in various ways depending on the material data (or standards) adopted in the calculation. The main considerations for the selection of the standards and material data are (a) the ease of using the data for calculation and (b) the extent of recognition of the standards in the market. However, it should be noted that the material creep properties from various standards have wide variation from heat to heat. The time to rupture prediction generally provides a guideline to help to establish the trend relative to failure and time of replacement and should not be used as an exact life calculation [25]. The common standards used for creep life calculation are:

- API 530 [26]

Although this standard indicates that the calculation is meant for refineries, the creep life calculation aspect is also applicable to static components in the energy sector. The creep model used in this standard is LMP, which is supplemented by the Welding Research Council (WRC) Bulletin 541 [27] (more details in Appendix B).

- API 579-1 [19]

The calculation procedure is applicable to components that are subject to steady state operation in the creep range which do not have crack-like flaws. Two types of material data for time-to-rupture calculation are introduced in the standard: creep rupture data in terms of LMP (similar to the ones indicated in API 530) and MPC Project Omega data. The latter does not include the effects of primary creep.

- PD 6525 [28]

The standard PD 6525 part 1 uses MHP, which is a linear time–temperature relationship for creep and stress rupture data [29]. The advantage of MHP to LMP is that MHP has two constants relating time to temperature (i.e., log(ta) and T_a_) as compared to only one constant in LMP (i.e., C). This might allow MHP to have better sensitivity to the time–temperature relationship that enables higher life prediction accuracy (more details in Appendix B).

- BS EN 12952-4 [30]

The focus of the calculation procedure, described in this standard, is the computation of time to rupture. Theoretical lifetime can be estimated using a diagram of creep rupture strength versus time to rupture.

6. Step 6—determine the accumulated creep damage consumed

As creep damage is sensitive to local stresses and temperatures, a small change in these factors will lead to significant variation in the rate at which creep damage accumulates [2,4]. Damage rules are developed to calculate the amount of life expended as a function of specific conditions and the two common rules are the sum time fractions and strain fractions, with the former being more widely used. Most creep life calculations are based on the Robinson life fraction rule (i.e., a type of sum time fractions), as shown in Equations (6) and (7). Some considerations should be noted when using life fraction rules: (a) they are generally valid for cases involving temperature changes and not stress changes; (b) cumulative fraction rule at failure depends on the nature of the existing degradation, ductility of material and order in which changes in stress and temperature are imposed, and it can be greater than, equal, or less than unity.

D = Σ (t_i_/t_ri_), (6)

D = Σ (ε_i_/ε_ri_), (7)

ti: the time spent under condition i (h);
t_ri_: the time to rupture under condition i (h);
ε_i_: the strain accumulated under condition i (%);
ε_ri_: the strain to rupture under condition i (%).

7. Step 7—determine the future creep damage at fixed time intervals

The future creep damage calculation is performed by repeating steps 1 to 5 for future operations. The time increments are required to be small enough to provide reasonable accuracy in the estimated creep life. An assumption of no change in the component’s operating parameters is used.

8. Step 8—estimate the residual creep life

The estimation is based on the accumulated creep damage of past and future operations and is computed according to Equation (8). The accumulated creep damage iteration calculation will stop when the total summation of creep life fraction consumed is 1.

Total creep life fraction consumed = Σ^N^_n = 1_(T^n^_op_/T^n^_rupture_) = 1. (8)

There also exist alternatives to the methods described above:

- Other time–temperature parameter models for calculations;
- A rough estimation can be performed based on the extent of microstructural degradation on the condition that in situ metallographic replication was conducted previously;
- If hardness data are available, an estimation based on these results can be performed using LMP methods.

#### 3.1.3. Inspection Plan Development

An inspection plan can be developed once RLA is completed and the susceptible areas are identified. The objective of the last stage of L1 assessment is to develop an inspection plan to establish the presence of creep damage (if any) on the susceptible components. The inspection plan generally includes the following information:

- Component to be inspected;
- Susceptible damage mechanisms;
- Parts of a component that are prone to certain damage mechanisms;
- Non-destructive technique to be used to address the type and extent of the damage.

### 3.2. Level 2

**Figure 3:**
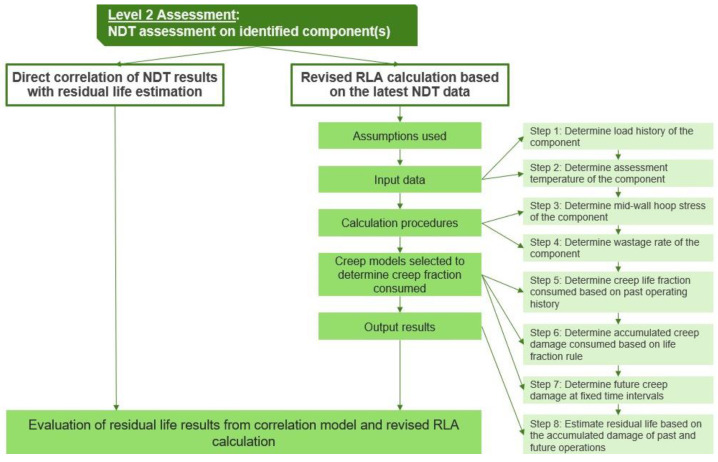
Flow chart on the steps for L2 approach in creep RLA.

Once the component is deemed to be susceptible to creep damage in L1 analysis, non-destructive testing (NDT) assessment (Figure 3) is recommended to be carried out on the components to establish the extent of creep damage. Thus, L2 analysis aims to evaluate the residual creep life on identified components from NDT results directly or from revised RLA calculations that are based on NDT results. Similarly to L1, the RLA calculation in L2 also comprises 8 steps, which are detailed in Section 3.1.

In most of the recommended practices and guidelines creep damage detection is generally split into two types [31,32,33]: techniques for detecting an early stage of creep damage, namely, stages 1 and 2, and techniques for detecting creep fissures or cracks, or stage 3.

Internationally recognized advanced techniques that other industry players are using to assist in creep life assessment and novel technologies that one can consider for creep damage detection are field metallographic replication, in situ hardness test, dimensional inspection, internal oxide thickness measurement/wall thickness measurement, advanced ultrasonic techniques, acoustic emission test, scanning force microscopy, and electromagnetic methods. Their creep detection capabilities, advantages, and disadvantages are summarized in Appendix A.

In the case that the evaluation in L2 demonstrates that a component sustained some extent of creep damage and an accurate residual life cannot be determined, it is recommended to proceed with a more detailed analysis of the component. This is particularly important when creep pores are observed, and significant wall thinning is detected. Thus, L3 analysis is proposed for more precise life predictions of the creep-damaged components.

### 3.3. Level 3

**Figure 4:**
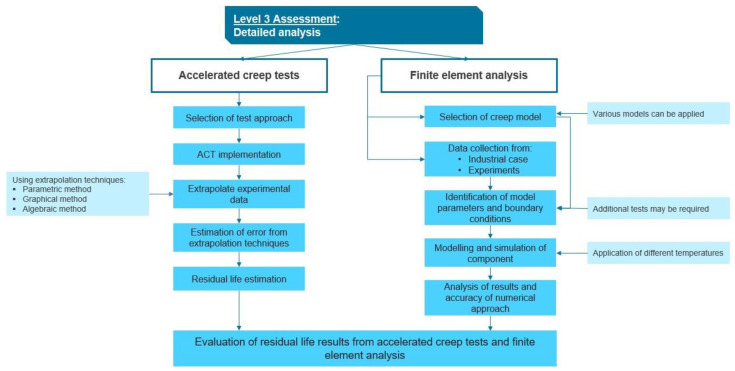
Flow chart on the steps for L3 approach in creep RLA.

L3 assessment aims to provide an amplified examination of components if L2 assessment is not sufficient to evaluate residual life. A flow chart of the proposed L3 is shown in Figure 4.

The L3 assessment is split into two parts: destructive accelerated creep tests (ACT) and finite element analysis (FEA). In the first part, destructive ACT, different test approaches and extrapolation of test results to obtain residual life estimation are explored. The second part, namely FEA, is proposed to complete the RLA protocol. Many parameters and service conditions affect the creep life of high-temperature equipment. Hence, numerical simulations have great potential due to the minimization of calculation and experimental time while providing a fast response.

#### 3.3.1. Accelerated Creep Tests

Destructive tests provide a direct measure of the current damage state of the material [34]. It is often the last resort of the residual life assessment for creep damage, as it requires the removal of material from the existing component. The test is often limited by the number of available samples and locations in which they can be taken.

There are various types of experimental creep tests available in the industries. The most used creep test for power industry is the uniaxial tensile ACT for RLA. The most employed rupture test methods are as follows [35]:

1. Both test temperature and/or applied stress are elevated over the in-service condition. This means that both temperature and applied stress parameters are different for all tests;
2. The test temperature is elevated while the applied stress is maintained constant for all tests at a level representative of the in-service condition (iso-stress rupture test).

The first difficulty that arises from ACT is that few material data of up to 100,000 h are available for even the established material [36]. Therefore, it is necessary to extrapolate from data obtained from much shorter tests (i.e., from 1000 to 10,000 h) for life prediction purposes. There are three main groups of extrapolation techniques [36]:

- Parametric method, which is the most used method for high-temperature components in the power industry;
- Graphical method (theoretical and empirical), but not widely employed;
- Algebraic method, although little advancement of this method for extrapolation of creep test data was observed over the years;

Three classes of parameters are categorized in the parametric method [36]: time–temperature (t, T), stress-modified (t, T), and time–stress (t, T) parameters. The first two parameters are closely related, and both represent the dominant part of the work conducted in the field. The extrapolation is usually performed using the time–temperature method.

#### 3.3.2. Finite Element Analysis

A numerical assessment of the residual life of components subjected to creep is proposed as the second part of L3. At the present moment, clients from the power industry start showing their interest in finite element modelling to obtain a rapid response for RLA, although, as an accurate complementary analysis to L2. In our methodology, FEA is seen as an independent tool that has the potential to do both: to replace time-consuming experimental creep tests in L3 and to serve as additional input to L2 analysis. In both cases, particular attention should be given to the definition of input data, boundary conditions, and selection of creep model. However, the complexity of the final numerical model is expected to be in line with objectives and the assessment level.

There exists a variety of creep damage models for components operating at various temperatures and stresses that can be used for FEA. They are categorized as empirical, phenomenological and physically based models [37,38]. The empirical models are most frequently used to estimate the RLA of components in the industry, which is related to their simplicity, rapidity of calculations, and interpretation of results. Nevertheless, these models have some limitations and assumptions. For instance, a component is supposed to have a stable material structure during creep deformation. Thus, the presence of cavitations, precipitations, changes of phase, changes in fracture mechanics, and other modifications may lead to less reliable results of RLA, errors, and overestimation of the long-term creep life [39,40]. Some of the widely used models are Larson–Miller [27,37,38,39,41,42], Manson–Haferd [37,38,42,43], Norton [37,44,45], Norton–Bailey [44], Omega [38,41,45,46], and others. The phenomenological models are less employed than the empirical ones due to their complexity. However, the research community was intensively developing and using these approaches for their better accuracy, especially in various damage, fatigue, and creep problems. These models rely on continuous damage mechanics (CDM) and are no longer based on data fitting. The principal difference is that the parameters in CDM models reflect various microscopic processes and account for both plasticity and creep. However, they require a large number of material constants [37]. Thus CDM models are not often used for industrial applications and yet are broadly applied for research purposes and some critical and complex industrial cases. Physically based models are believed to provide the best accuracy. They are based on creep constitutive equations from microstructural features varying over time, such as dislocation and interparticle spacing mechanisms, and temperature [38]; yet these types of models are very complex to implement for industrial needs [47]. Numerous empirical and phenomenological creep models are integrated into FEA software packages. The validation of the numerical RLA of a component in the secondary creep is underway in the final phase.

## 4. Case Study and Discussion

An application of the proposed three-level RLA methodology to an industrial case study is described in this section. For confidentiality reasons, the name of the power plant, the location of the analyzed tube, and some of the results cannot be revealed.

Our team performed RLA on a superheater tube from a sub-critical boiler, which was subjected to regular inspections. The details of the tube construction, design, and operating parameters are:

- Material: ASTM A213 Grade T91;
- Outer diameter: 60.33 mm;
- Design thickness: min. nominal 5.537 mm, min. allowable 4.037 mm;
- Design temperature: 580 °C;
- Operating pressure: 180 bars;

The available inspection data for L1 assessment are summarized in Table 2.

### 4.1. Level 1

The preliminary assessment, based on the accessible information, was carried out in L1. For L1, only the inspection records for periods 1 and 2 are available as summarized in Table 2. Hence, the wastage rate, the hoop stress and the tube metal temperatures were estimated according to the calculation approach, described in steps 1–4 in Section 3.1.2., and the data for both periods. The results for periods 1 and 2 are summarized in Table 3.

The time to rupture, the creep life fraction consumed, and the estimated residual life were calculated according to steps 4–8 from Section 3.1.2. The results are summarized in Table 4. All the calculations in steps 1–8 were performed using an internally developed Python-based code.

Two methods for computation of average and minimum properties are used: LMP creep model from the standard API 530 and MHP creep model from the standard PD 6525 (please refer to Appendix B for more details) [26,28]. The estimated time-to-rupture and residual life results are of a similar range for both approaches. The estimated residual life of the tube based on LMP and MHP methods were 9.3 and 10.9 years, respectively. This indicated that creep damage was a cause for concern, and therefore, the tube metal temperature and wastage rate should be verified. In order to have more accurate results, L2 assessment was recommended.

### 4.2. Level 2

A shutdown inspection was performed on the tube panel. The updated inspection record of the tube with the latest findings is summarized under period 3 in Table 2. The recalculated wastage rate, the hoop stress and the tube metal temperature can be found in Table 3 under period 3.

With an operating period of 120,000 running hours, the oxide thickness was 0.42 mm. During the shutdown, the measurement data revealed that tube sustained some wall loss, i.e., 1.14 × 10^−5^ mm/h in comparison to 6.67 × 10^−6^ mm/h. This would have a significant impact on the estimated residual life calculated in the L1 assessment. Therefore, a re-calculation was performed to evaluate how the increase in the wastage rate affects the residual life estimation. The summary of the revised residual creep life, based on the latest on-site measurement data, is shown in Table 5.

The calculation trend in the L2 assessment is similar to the L1 assessment, in which the estimated time-to-rupture results that correspond to 120,000 running hours are of the same range. The LMP and MHP approaches provide similar residual life estimations with a minimum life of 5.1 and 4.4 years, respectively, and average life of 8.5 and 10.1 years, respectively.

### 4.3. Level 3

During the shutdown inspection, a portion of the tube was sectioned for ACT tests in L3 assessment. The tube section was then machined into three test specimens. Prior to ACT, laboratory analysis was performed on the tube section to obtain the wall thickness, the outer diameter, and the internal oxide thickness. Based on the new oxide thickness, the recalculated tube metal temperature was slightly higher than 583 °C.

Iso-stress rupture tests were carried out and extrapolation was performed using LMP and MHP. The newly estimated time-to-rupture results are higher than those calculated in L2. The deviation of time-to-rupture extrapolated results with LMP and MHP are very similar. The residual creep life of the tube was also higher in L3 than in L2 calculations.

### 4.4. Discussion

A preliminary L1 assessment provides insights on the estimated residual creep life of the tube if the wastage rate and oxidation rate remain the same as the ones calculated in period 2. As the minimum residual creep life is less than 5 years, it is recommended to perform another round of assessment to verify if the creep damage worsened at the end of period 3.

Based on the L2 calculation using actual site results, the estimated creep life at the end of period 3 was similar to the calculated residual life at the end of period 2. This suggested that the creep damage in the boiler tube did not worsen when it was in service between periods 2 and 3. However, in view of its low calculated residual creep life, a portion of the tube was removed for ACT in L3 assessment.

The extrapolated results from L3 assessment, using the material properties data extracted from ACT of the tube sample, reveal higher residual creep life values than L2 calculation. This indicates that the creep-damaged boiler tube is safe to run for more than 5 years (i.e., the residual creep life value calculated from L2 assessment).

It is expected to observe deviation in the residual creep life values between L2 and L3 assessments. The calculated residual life in L2 assessment is based on the material properties from virgin tubes that are published in standards, while the material properties used in L3 assessment were directly obtained from ACT of the sampled creep-damaged tube. Hence for L2 assessment, a safety factor is incorporated in our calculation in order to be on the conservative side.

## 5. Conclusions

After exploring various types of methodologies and approaches adopted by the power industry for components prone to creep, the general approach for residual life assessment is introduced to assess the likelihood of creep damage and to give recommendations if damage is detected. The proposed methodology is based on the numerous approaches formerly adopted by international bodies, recent developments in the field, and Cetim-Matcor’s experience. The methodology is composed of three levels:

1. L1 assessment is expected to provide a preliminary overview of the susceptible areas for creep damage based on design and operating history, as well as past inspection data.
2. L2 assessment is intended to establish the extent of creep damage if a component is detected as prone to creep in L1. It is based on the results from non-destructive testing.
3. L3, or detailed analysis, of components is proposed for cases when a more accurate life prediction, compared to L1 and L2, is necessary. It is especially the case if components already have some extent of creep damage, for instance, identified creep pores or diametrical strain. Accelerated uniaxial creep tests are meant to provide relatively precise results using extrapolation methods, whereas finite element analysis is aimed at identification of thermo-mechanical behavior over time and of structural durability in complex cases with the minimization of time of assessment in comparison with accelerated testing.

The three-level methodology was applied to a real industrial case study for demonstration and validation purposes. It was found that there were some differences in the residual life results calculated in L2 and L3 using data from accelerated creep tests. This suggests that more studies are required to reduce the uncertainty to improve the accuracy of the calculated and extrapolated results.

## Figures and Tables

**Table 1:** Required information for L1.

Information Gathered	Mandatory/ Optional	Remarks
Design and operating data	Mandatory	These parameters are readily available from clients most of the time.
Equipment data	Mandatory	The important parameters required for calculation are outer diameter, thickness, and material specification. For L1 calculation, the above data will be extracted from the inspection records, if available. If there are no inspection records, the parameters for calculation will be extracted from the design data of the equipment.
Maintenance records	Mandatory	
Inspection records	Mandatory	
Monitoring records	Optional	-

**Table 2:** Inspection records for the tube.

Period	Running Hours	Wall Thickness	Oxide Thickness	Hardness
1	55,000 h	5.5 mm	0.22 mm	220 HV
2	85,000 h	5.2 mm	0.32 mm	210 HV
3	120,000 h	4.8 mm	0.42 mm	200 HV

**Table 3:** Assessed information for the tube.

Period	Running Hours	Oxidation Rate	Wastage Rate	Hoop Stress	Tube Metal Temperature
1	55,000 h	1.91×10^−6^ mm/h	2.49×10^−6^ mm/h	91.0 MPa	568 °C
2	85,000 h	1.59×10^−6^ mm/h	6.67×10^−6^ mm/h	94.8 MPa	577 °C
3	120,000 h	1.36×10^−6^ mm/h	1.14×10^−5^ mm/h	103.5 MPa	583 °C

**Table 4:** Residual life calculation results for L1 from various empirical calculation models.

Parameters	Average Properties		Minimum Properties	
	API 530 (LMP)	PD 6525 (MHP)	API 530 (LMP)	PD 6525 (MHP)
Time to rupture ^1^	4.96 × 10^5^ h	6.71 × 10^5^ h	1.49 × 10^5^ h	1.16 × 10^5^ h
Creep life fraction consumed ^1^	0.041	0.031	0.138	0.183
Estimated residual life	82,000 h 9.3 years	96,000 h 10.9 years	46,000 h 4.5 years	40,000 h 4.5 years

^1^ Till period 2.

**Table 5:** Residual life calculation results for L2 from various empirical calculation models.

Parameters	Average Properties		Minimum Properties	
	API 530 (LMP)	PD 6525 (MHP)	API 530 (LMP)	PD 6525 (MHP)
Time to rupture ^1^	1.30 × 10^5^ h	1.99 × 10^5^ h	3.91 × 10^4^ h	6.52 × 10^2^ h
Creep life fraction consumed ^1^	0.188	0.134	0.624	0.801
Estimated residual life	75,000 h 8.5 years	89,000 h 10.1 years	45,000 h 5.1 years	39,000 h 4.4 years

^1^ Till period 3.

## Data Availability

Data not available due to legal restrictions.

## Appendix A

Review of internationally recognized NDT techniques:

- Field metallographic replication (FMR)

Creep detection capability: FMR can detect early stage creep damage in terms of creep pores/cavities.

Advantages: damage evaluation on the microstructure can be carried out without sectioning the component; can reveal the extent of creep-related damage; fast, inexpensive, and can be used in many locations [4,48].

Disadvantages: can only evaluate the surface condition of the component; does not provide volumetric information of the defects detected [4,33,48].

To evaluate remnant creep life, base metal damage and creep cavitation calculation models were developed based on FMR results.

The procedure for producing and evaluating replicas is described in ASTM E1351, whereas the guidelines from API 579 can be used to estimate the expended creep life based on creep pores/cavities.

- In situ hardness test

Creep detection capability: the test does not detect creep damage, however, a correlation with the residual creep life prediction can be carried out using a calculation model based on LMP [18].

Advantages: simple and low-cost technique; rapid data acquisition; and can be performed on various components [32].

Disadvantages: careful surface preparation is critical; prone to have widely scattered data; sensitivity to local microstructural variation; strain-softening effects; and uncertainties on the initial hardness of the material [4,32].

API 579 provides recommendations on the component susceptibility to creep damage based on hardness results.

- Dimensional inspection

Creep detection capability: the test provides indicative signs of creep damage based on the extent of swelling/bulging.

Advantages: monitoring of gross changes such as component swelling and other dimensional changes [49].

Disadvantages: if the original dimension is unknown, the changes in the dimensions cannot be determined with confidence; no provided indications of severely creep-damaged areas or areas that sustained localized creep strains; and no correlation between the extent of swelling and remnant creep life estimation [49].

- Internal oxide thickness measurement/wall thickness measurement

Creep detection capability: this measurement does not detect creep damage directly. However, it provides metal temperature estimation, which is critical for remnant creep life estimation.

Advantages: a reliable technique with reproducible results; simple to perform on site [18].

Disadvantages: minimum detection limit of 0.15 mm for the oxide thickness measurement; the method is only applicable to boiler tubes due to the limited availability of transducer/probes to measure thicker pipes or headers [18].

- Advanced ultrasonic techniques (UT) (time-of-flight diffraction test (TOFD), phased-array ultrasonic test (PAUT), and electromagnetic acoustic transducer (EMAT) test).

Creep detection capability: can detect early stage creep damage in terms of creep pores/cavities [50,51,52,53,54].

Advantages

TOFD: accurate sizing capability; quick to set up and perform the inspection; rapid scanning with imaging; and highly sensitive to all flaw types [55,56,57].

PAUT: simplified inspection and interpretation; increase coverage for flaw detection; more reliable results; and faster inspection speeds as compared to the conventional UT [58,59,60]. The latest PAUT technology allows the detection of clustered cavities/pores.

EMAT: non-contact technique, can perform the inspection while the component is in service; not affected by the surface condition of the component; no couplant required; and easy sensor deployment [61,62,63,64].

Disadvantages

TOFD: not suitable for coarse grain material; requires crack edge to be sharp for distinct diffracted echoes; and dead zone for defect detected under the surface [55,56,65].

PAUT: not recommended for surface cracks, metal fatigue, bolt hole or tubing inspection; requires a qualified and trained professional to perform and interpret the results; and more expensive as compared with conventional UT [58,59,60].

EMAT: applicable for materials that conduct electricity; consumes more power than other UT techniques, as it requires very high power to generate the sound; and bulky magnets that can be difficult to use on the component [61,62,63,64].

- Acoustic Emission Test (AET)

Creep detection capability: can locate the defect within the material while the components are under operation/stress [66].

Advantages: provides real-time data; a structure can be monitored globally; can be used in environments with high pressure and/or temperature; the structure/component can be assessed under operational conditions; can be conducted remotely; and can detect defects in materials that are hard to be detected by other NDT methods [67,68].

Disadvantages: can only provide a qualitative estimation of the extent of damage, i.e., able to indicate the location of a defect but unable to provide more details on the defect features; cannot detect defects that do not change with time; difficult to interpret the data as the AET signals is generally weak compared to the background noise; and it involves the use of complex and expensive hardware and software [67,68].

There are various standards and industry organizations that developed regulations for AET. The relevant ones for power industry are mainly from EPRI [69,70,71].

- Scanning Force Microscopy (SFM)

Creep detection capability: SFM can detect early creep damage, namely, defects of the sizes of 0.01–0.1 µm, assess their depth as well as provide quantitative results of the section analysis.

Advantages: it is portable and can be used on-site, linked to a laptop, and instantaneously transmit images by internet; it attaches to on-site components on a flat or curved surface using straps and/or magnets; surface preparation equivalent to replication, i.e., 1 μm polish and etch; spatial resolution comparable to a scanning electron microscope (SEM), but does not require the use of vacuum equipment; 3D scanning provides surface roughness and cavity depth volume data; can scan metallic and non-metallic materials/components; and can provide real-time capture of data and imagery; can be used to assess certain material properties [72,73,74].

Disadvantages: cannot detect the defects that change with time; SFM is most effective for P91 and P92 or similar materials; required component polishing and etching; magnification range is lower than that of conventional SEM; and the technique remains relatively new and not widely used [72,73,74].

- Electromagnetic methods (Eddy current testing, remote field testing, near-field testing, surface Eddy current testing, pulsed Eddy current, internal rotary inspection system, magnetic flux leakage testing, magnetic particle inspection).

Creep detection capability: can detect and size degradation within a material; methods can be classified as surface testing methods.

Advantages (on the example of Eddy-current testing): sensitivity to surface defects, defect detection through several layers and surface coatings; accurate conductivity measurements; can be automated; little pre-cleaning required; and portable [75,76,77].

Disadvantages (on the example of Eddy-current testing): sizing of defects may not be very precise; very susceptible to magnetic permeability changes; only effective on conductive materials; does not detect defects parallel to surface; not suitable for large areas and/or complex geometries; signal interpretation required; no permanent record (unless automated) [75,76,77].

## Appendix B

1. API 530 Calculation of Heater tube Thickness in Petroleum Refineries

The LMP creep model is as follows.

LMP(σ) = (T + 460)(C + log_10_[L_d_]) (A1)

LMP = A_0_ + A_1_log_10_[σ] + A_2_(log_10_[σ])^2^ + A_3_(log_10_[σ])^3^. (A2)

σ: hoop stress (ksi);
C: Larson Miller Constant with minimum and average values provided for each material specification;
T: assessment temperature (°F);
L_d_: time to rupture (h);
A_0_, A_1_, A_2_, A_3_: coefficients depending on material specification of component. The coefficient data are provided in the WRC bulletin 541 [27].

The creep rupture curves for respective materials are intended for use where the design stress is greater than 6.9 MPa. It shall not be used to determine rupture allowable stresses for temperatures higher than the limiting design metal temperature. The curves can give inaccurate rupture allowable stress for a tube life of less than 20,000 h or greater than 200,000 h.

2. PD 6525 Part 1: Elevated Temperature Properties for Steels for Pressure Purposes Part 1: Stress Rupture Properties

MHP was introduced in 1953 to address the inaccuracy of the LMP resulting from the suggestion of a fixed C for metallic material. It is important to note that there was advancement in the LMP method over the past decades, in which C values vary with different materials. For MHP, it is assumed that the logarithm of the time varies linearly with the temperature at a constant initial stress. This is illustrated in the equations as follows.

MHP(σ) = (log(t) − log(ta))/(T − T_a_)^r^ (A3)

MHP(σ) = a + b(log(σ)) + c(log(σ))^2^ + d(log(σ))^3^ + e(log(σ))^4^ (A4)

T: Temperature (K);
t: time to rupture (h);
σ: hoop stress (MPa);
r: a temperature exponent provided in Appendix B of the standard PD 6525 Part 1 [28];
T_a_, log(ta), a–e: constants provided in Appendix B of the standard PD 6525 Part 1 [28].
